# Analysis of gadolinium oxide using microwave-enhanced fiber-coupled micro-laser-induced breakdown spectroscopy

**DOI:** 10.1038/s41598-023-32146-x

**Published:** 2023-03-24

**Authors:** Yuji Ikeda, Joey Kim Soriano, Hironori Ohba, Ikuo Wakaida

**Affiliations:** 1 i-Lab., Inc., #213 KIBC Bldg., 5-5-2 Minatojima-Minami, Chuo, Kobe, 650-0047 Japan; 2 Collaborative Laboratory for Advanced Decommissioning Science, Japan Atomic Energy Agency (JAEA), 2-4 Shirakata, Tokai-mura, Naka-gun, Ibaraki 319-1195 Japan; 3 Quantum Beam Science Research Directorate, National Institutes for Quantum Science and Technology (QST), 2-4 Tokai-mura, Naka-gun, Ibaraki 319-1106 Japan; 4 Collaborative Laboratory for Advanced Decommissioning Science, Japan Atomic Energy Agency (JAEA), 790-1 Motooka, Tomioka-machi, Futaba-gun, Fukushima 979-1151 Japan

**Keywords:** Spectrophotometry, Laser-produced plasmas

## Abstract

We report on the analysis of pure gadolinium oxide (Gd_2_O_3_) and its detection when mixed in surrogate nuclear debris using microwave-enhanced fiber-coupled micro-laser-induced breakdown spectroscopy (MWE-FC-MLIBS). The target application is remote analysis of nuclear debris containing uranium (U) inside the Fukushima Daiichi Nuclear Power Station. The surrogate nuclear debris used in this study contained gadolinium (Gd), cerium (Ce), zirconium (Zr), and iron (Fe). Ce is a surrogate for U, and Gd_2_O_3_ is an excellent hazard index because it is incorporated into some fuel rods. Gd detection is essential for assessing debris prior to the retrieval process. Surrogate debris was ablated by an 849 ps 1064 nm micro-laser under atmospheric pressure conditions while a helical antenna propagated 2.45 GHz 1.0 kW microwaves for 1.0 ms into the laser ablation, which was then characterized by a high-speed camera and high-resolution spectrometers. The results showed that microwave-induced plasma expansion led to enhanced emission signals of Gd I, Zr I, Fe I, Ce I, and Ce II. No self-absorption of Gd emissions was evident from the detection limit calibration graphs. Moreover, microwave irradiation decreased the standard deviations of the Gd and Ce emissions and lowered the Gd detection limit by 60%.

## Introduction

During the decommissioning of nuclear fuel debris at the Fukushima Daiichi Nuclear Power Station, the storage and disposal of radioactive materials have become major issues^1^. Sorting the complex mixture of concrete columns, steel barriers, fuel rods, and molten nuclear fuel is hampered by a highly radioactive environment exceeding 70 Gy/h^2^. Although it is possible to explore the interior of the reactor with a high-resolution camera, quantitative analysis is required to understand the nuclear contamination level of each piece of debris. X-ray fluorescence (XRF) measurements have also been researched for in-core detection^3^. However, the radiation durability of fiber-coupled (FC) laser-induced breakdown spectroscopy (LIBS), which is able to withstand 800 Gy/h for 2 h, confirms guaranteed in-situ operation^4–7^.

The presumed composition of the nuclear fuel debris includes uranium oxide (UO_2_) from the nuclear fuel, zirconium (Zr) from the cladding, stainless steel (Fe, Ni, Cr) from the surrounding structural material, and gadolinium oxide (Gd_2_O_3_), which was incorporated into some of the fuel rods^1,2,8^. Gd_2_O_3_ is used to control fuel reactivity, as gadolinium (Gd) acts as a burnable absorber of thermal neutrons with the aid of the high neutron absorption cross-sections of ^155^Gd and ^157^Gd isotopes^8,9^. Gd is, therefore, an excellent hazard index for each piece of debris; thus, the measurement of the relative abundance of Gd in the debris is essential. However, because of the high radioactivity inside the reactor vessel, the nuclear fuel debris is currently inaccessible and is yet to be identified. Thus, there is a compelling need for methods to remotely analyze the debris and detect the above elements in a high-radiation field.

Because the actual fuel debris is not available as a sample, FC-LIBS measurements were performed using surrogate nuclear debris synthesized from mixed oxide materials containing Gd, Zr, Fe, and cerium (Ce). Ce is a surrogate for uranium (U) because of the similarities in its electronic structure^9,10^. Detection of Gd using LIBS in such complex materials is difficult because of the presence of dense spectral lines from rare-earth elements that strongly interfere with each other.

LIBS uses laser-induced plasma optical diagnostics for the elemental analysis of complex mixtures, with minimal to no sample preparation^11,12^. The technique has been used to successfully detect and analyze various samples containing U^7,13–15^. U in its naturally occurring form can be enriched into various atomic fractions of fissile materials for use in nuclear reactors. A recent review of LIBS applications to U plasmas^16^ listed the advantages of using LIBS for the identification of nuclear materials, including the long-range rapid detection of nuclear material isotopic composition. However, the radiation-induced attenuation of light transmission limits the application of LIBS; thus, micro-lasers have been developed^9^.

A micro-laser is composed of composite ceramics pumped by a quasi-continuous wave (CW) diode laser, generating a sufficiently intense laser pulse for ablation and plasma formation on the target^17,18^. Several advantages of using micro-lasers were listed in the report on the development of the micro-laser^9^, including a disposable low-cost ceramic probe, which is essential for in-core nuclear detection applications. However, remote analysis under high radiation levels requires more than 50 m of optical fiber from the laser source to the detection site, which can attenuate the measured signal emission.

Microwave-enhanced LIBS (MWE-LIBS) overcomes the emerging challenges of the in-core detection applications of LIBS through enhanced emission^15,19–32^. As the microwave propagates into the laser-induced plasma, the excitation and recombination of atoms are driven into a cyclic process by the acceleration of free electrons. The expanding plasma increases the microwave absorption, which further increases the collisional activities of electrons, excited atoms, and surrounding air molecules. The result is a high-volume nonequilibrium plasma with lifetimes that last longer than the microwave duration. In turn, the signal-to-noise ratio (SNR) increases several hundred times owing to the enhancement of the emission intensity, which was previously reported for various targets such as aluminum oxide (Al_2_O_3_), lead (Pb) plates, copper (Cu) metal, and Zr metal^20–22,26^.

This study investigates the combined effects of micro-lasers and microwaves on the analysis of Gd_2_O_3_ in various forms, both pure and mixed in surrogate nuclear fuel debris. A high-speed camera and high-resolution spectrometers were used to characterize the plasma dynamics, emission enhancement factors, SNR, and limit of detection (LOD) of Gd. In addition, image analysis of the plasma formation was performed to measure the spread of plasma with and without microwaves, and to measure the temporal change of the plasma region. We also aimed to identify the effect of microwaves on the self-absorption of the Gd line in the LOD calibration curve and to clarify the effect of microwaves on LOD quantification.

## Materials and methods

### Ceramic micro-laser

**Figure 1:**
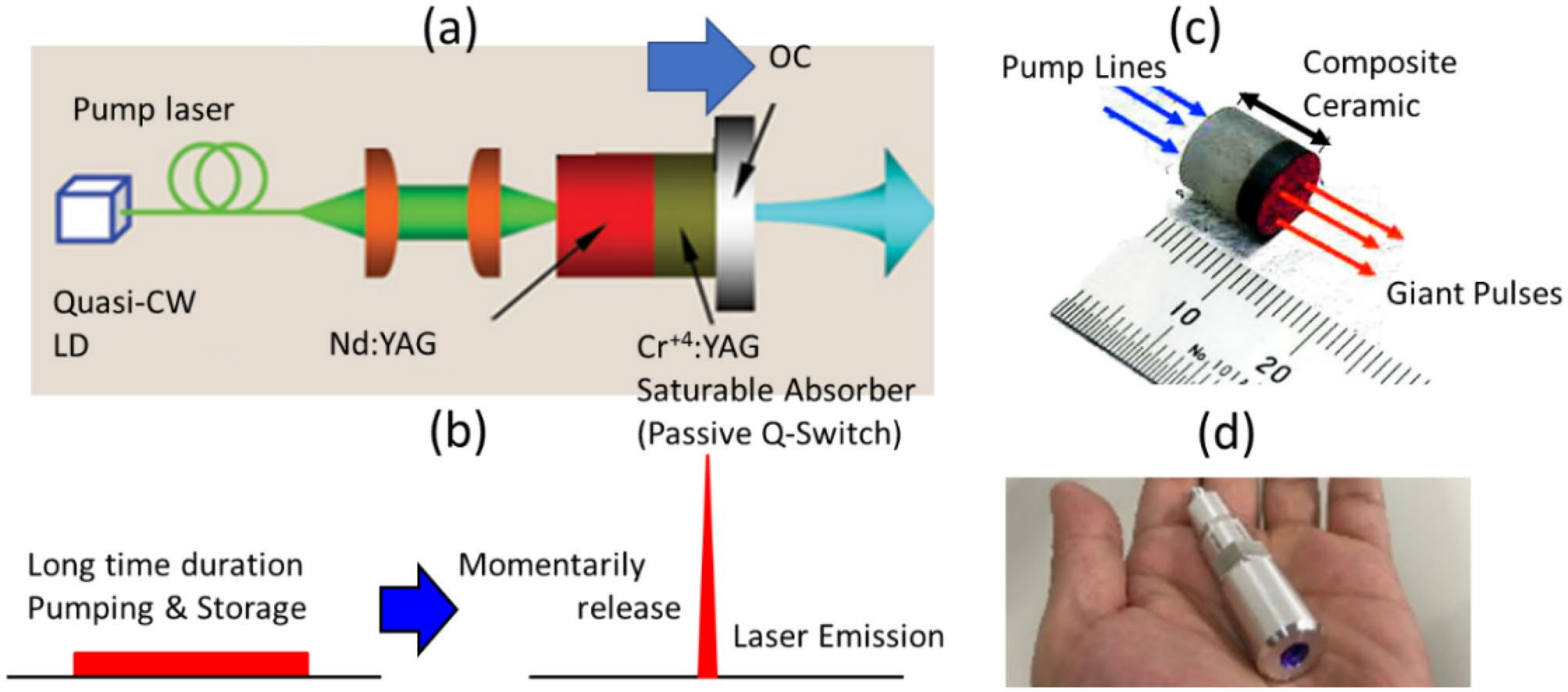
Micro-laser. (**a**) Illustration and (**b**) operating schematic of the monolithic Nd:YAG/Cr:YAG composite ceramic quasi-continuous wave (CW) laser diode (LD) micro-laser, along with actual images of the (**c**) ceramic composite and (**d**) stainless steel container.

Figure 1a and b show the operation schematics of the micro-laser, which consists of a monolithic Nd:YAG/Cr:YAG composite ceramic (Konoshima Chemical/Baikowski Japan, Japan). The Nd:YAG serves as a gain medium that is end-pumped by an 808 nm FC quasi-CW laser diode (JOLD-120-QPXF-2P iTEC; JENOPTIK, FRG). The laser diode produced a maximum output power of 200 W at a current of 120 A with a power supply (PS; PLWB168; UNITAC, Japan). Cr:YAG acted as a saturable absorber, enabling passive Q-switching within the composite without additional devices. Through pumping and storage (> 60 μs), the laser emits instantaneous laser pulses with 1.0 mJ laser energy, 849 ps pulse width, and 1064 nm wavelength. Figure 1c and d show actual images of the composite ceramic and its stainless-steel container. More information about the micro-laser can be found in reference^9^.

### Preparation of the surrogate nuclear debris

Pure Gd_2_O_3_ (Nilaco, Tokyo, Japan) was readily available in solid form. The surrogate nuclear debris was synthesized from powder mixtures of Gd_2_O_3_, CeO_2_, Zr_2_O_3_, and Fe_2_O_3_ (Oxide Powders, Rare Metallic, Tokyo, Japan). The predicted mass fractions for the nuclear fuel debris are based on references^8,9^. Each mixture (0.5 g) was compressed under hydrostatic pressure (10 kN) into a pellet with a diameter of 8 mm and sintered at 1375 °C for 5 h. Decreasing concentrations of Gd_2_O_3_ from 0.1 to 1.0% were essential for LOD measurements. The mass fractions of Ce, Zr, and Fe were fixed at 42.9 wt%, 23.0 wt%, and 7.9 wt%, respectively.

### Experimental set-up

**Figure 2:**
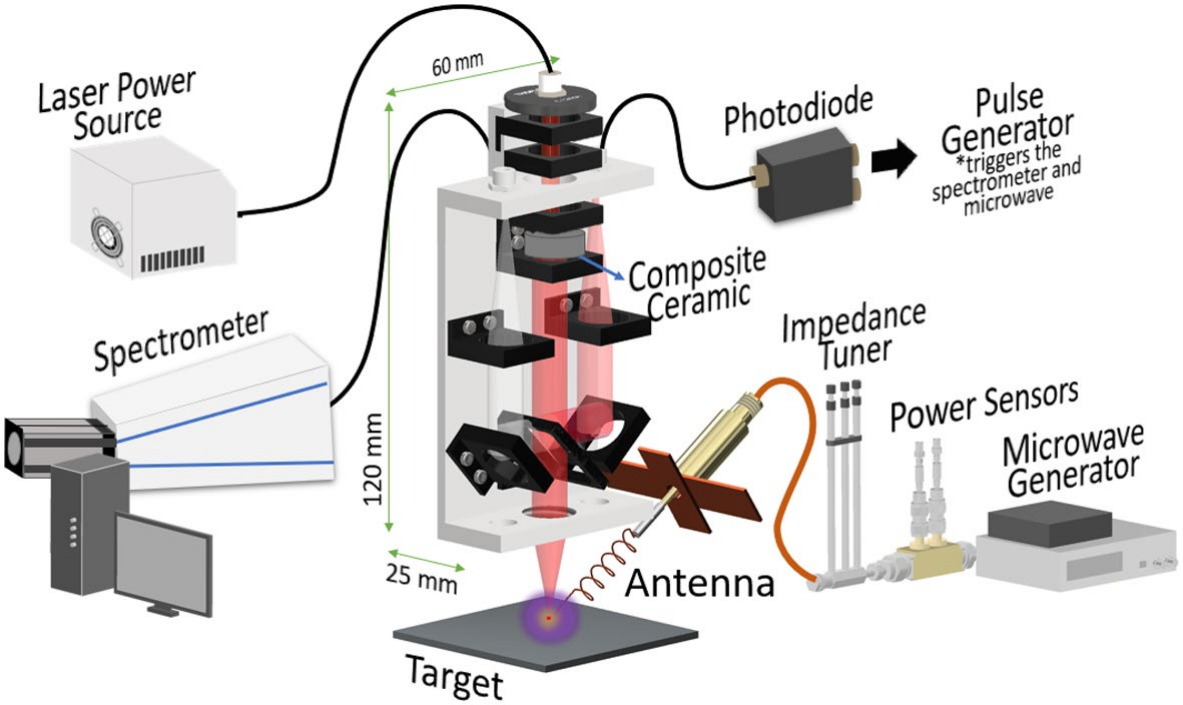
Schematic diagram of microwave-enhanced fiber-coupled micro-laser-induced breakdown spectroscopy (MWE-FC-MLIBS) using a ceramic micro-laser. The ceramic micro-laser composed of monolithic Nd:YAG/Cr:YAG is in a 60 nm × 120 nm × 900 nm aluminum housing. The ablation is coupled with a 2.45 GHz microwaves transmitted using a helical coil antenna with cross plate reflector.

The experimental setup for MWE-FC-MLIBS is shown in Fig. 2. A power supply that contains the laser diode transmits an 808 nm quasi-CW pulse into the composite ceramic through the optical fiber. A 60 nm × 120 nm × 900 nm aluminum casing housed the composite ceramic and optical elements. The laser output of the composite ceramic was transmitted to the beam splitter, which had a dichroic filter on the other side. The reflected light was transmitted to the photodiode with electrical pulses used to trigger the pulse generator. The final laser beam output of 1.0 mJ laser energy, 849 ps pulse width, and 1064 nm wavelength was focused into the target using a 25 mm focusing lens. The laser-induced emission was collected using a 600 µm optical fiber.

Two types of spectrometers with different spectral resolutions were used. An echelle-type spectrometer (ME5000, Oriel, Andor, UK) with λ/5000 resolution effectively analyzed the emission spectrum of pure Gd_2_O_3_ from 250 to 900 nm. Analysis of the surrogate nuclear debris was performed using another echelle-type spectrometer (Aryelle 400, Lasertechnik Berlin, Germany) with a very high spectral resolution of λ/50,000, which is capable of analyzing the surrogate nuclear debris. The microwave generator supplied the 2.45 GHz microwaves to the helical coil with cross-reflector plates^22^. The reflected power was minimized by an impedance tuner and monitored by power sensors.

### Characterization of the laser-induced plasma emission

An ultrafast camera (Fastcam SA-Z, Photron, UK) was used to visualize the plasma formation with the same synchronized signal as the laser firing. At 100,000 frames per second with an exposure time of 10 μs, a 720 × 380 pixel^2^ measurement area was captured. In all experimental conditions, a Tamron 180 mm AF macro lens (Saitama, Japan) was fixed at an aperture of F/3.5 and a zoom ratio of 1:10.

A straightforward characterization of the effects of microwaves on the laser-induced plasma is based on the intensity enhancement factor (IEF). The IEF is expressed by Eq. (1), which is obtained by taking the ratio of the intensity ${I}_{\uplambda ,\mathrm{MW}}$ of the emission measured using MWE-FC-MLIBS at wavelength $\uplambda$ and the corresponding emission intensity ${I}_{\uplambda ,\mathrm{LIBS}}$ for the standard LIBS without microwaves.

1 $$\mathrm{IEF }={I}_{\uplambda ,\mathrm{ MW}}/{I}_{\lambda ,\mathrm{LIBS}}$$

Another parameter that is affected by microwaves is the SNR, which is the ratio of the emission intensity to the standard deviation of the background signal. The SNR is measured as the height of the peak *I*_peak_ minus the average background signal *I*_BG,average_, which corresponds to the signal *H*. The signal *H* is divided by the standard deviation of the background noise signal *σ*, as shown in Eq. (2).

2 $$\mathrm{SNR}=H/3\sigma$$

In the detection of Gd, the LOD was derived from the slope of calibration curves (*S*) and the standard deviation (*σ*) of the blank solution using Eq. (3).

3 $$\mathrm{LOD}=3\sigma /S$$

## Results

### Effects of microwaves on the laser-induced plasma

**Figure 3:**
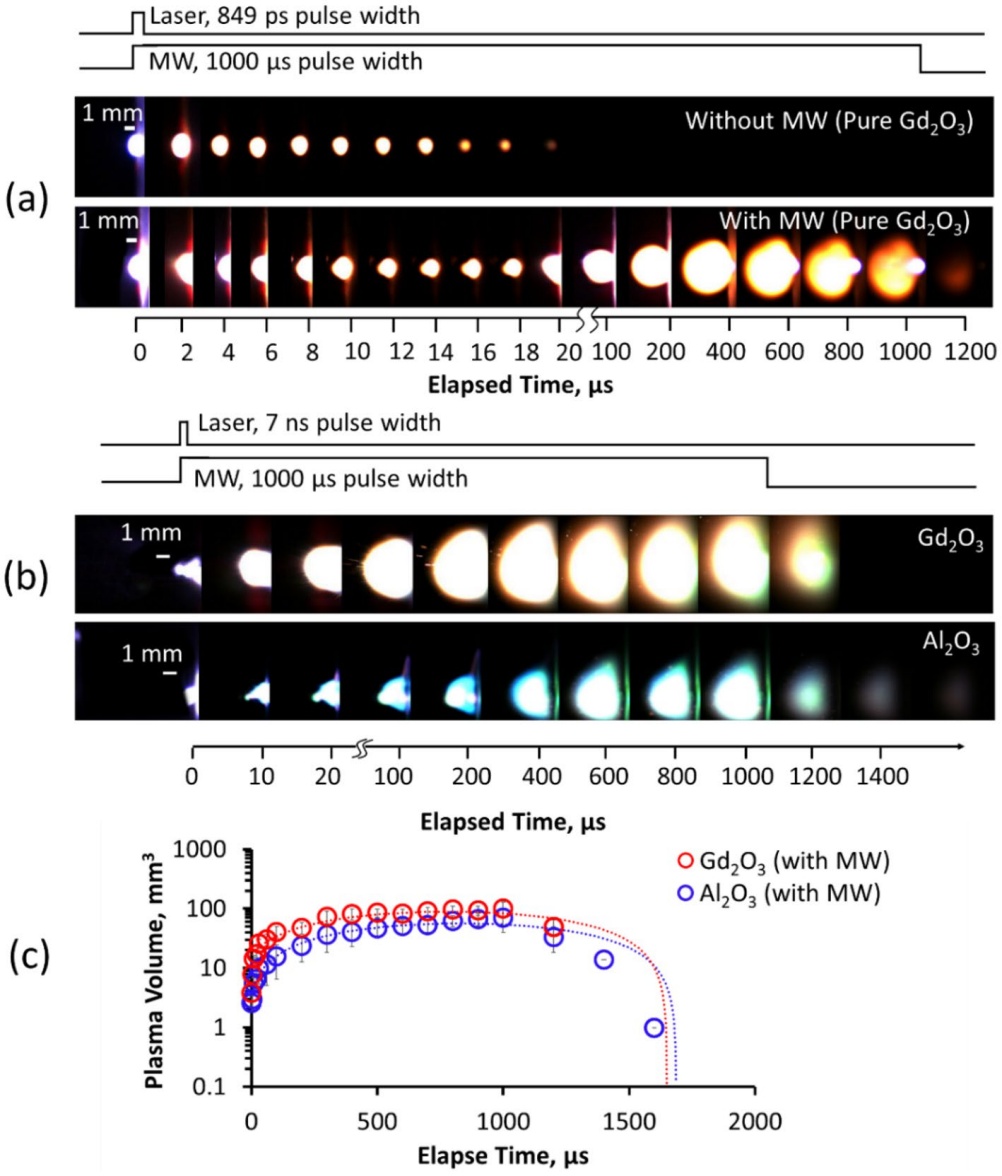
Visualized plasma formation of (**a**) pure Gd_2_O_3_ using MWE-FC-MLIBS and the standard micro-laser. The well-studied (**b**) plasma formation and the (**c**) corresponding plasma volume approximations on Al_2_O_3_ using microwave-enhanced nano-second ablation was also compared with pure Gd_2_O_3_.

Laser-induced plasma formation on the pure surface of the Gd_2_O_3_ sample with and without added microwave energy is shown in Fig. 3a with the corresponding plasma volume measurements in Supplementary Fig. 1. The plasma decreased gradually using a 1.0 mJ laser energy, and the emission lasted for 20 µs without the microwave. Using the same laser energy, expansion of the emitted laser ablation was observed to last until 1200 µs by transmitting 1.0 kW microwaves for 1.0 ms into the ablation area. The laser-induced plasma initially shielded the microwaves from 0 to 20 µs owing to its high density and collisional frequency of the electrons and neutral atoms^15^. As the impact of the laser energy diminished, the absorption of microwaves into the plasma increased, increasing electron mobility. The acceleration of the electrons ultimately affected the cyclic re-excitation and recombination of the Gd atoms, resulting in increased emission. Beyond 200 µs, the plasma and air interaction intensify as the contact between the plasma and the surface was reduced. The plasma exponentially expired after 1.0 ms after the microwave duration ended.

The plasma formation on pure Gd_2_O_3_ was also compared with that on Al_2_O_3_ and results show that the microwave expansion has the same dynamics (Fig. 3b,c). However, the plasma expands faster using pure Gd_2_O_3_, and the plasma is almost constant from 200 to 1000 µs. The plasma shielding of the microwave propagation was overcome sooner after 10 µs. The blocking of the microwave propagation prevented the interaction of the microwaves with the inner layer of the plasma, which was usually observed at approximately 10 µs^7,15,21^. As the microwaves started to interact with the laser-induced plasma, the plasma spread into the surrounding air and away from the sample.

### Spectroscopic measurement of pure Gd_2_O_3_

**Figure 4:**
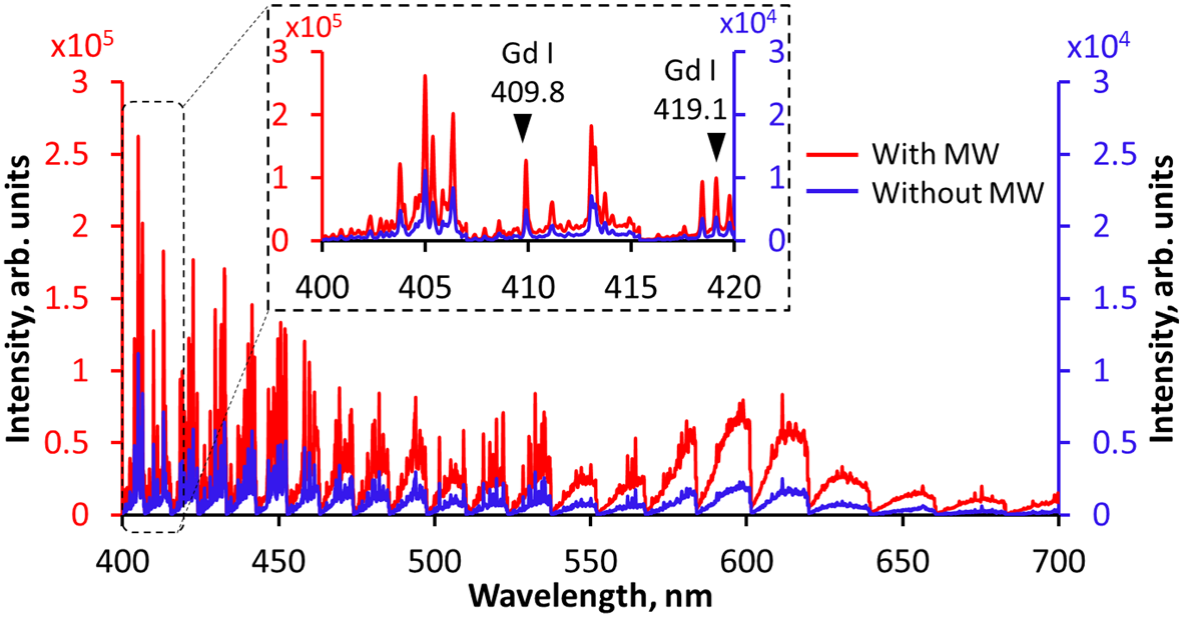
Emission spectra of 99.9% pure Gd_2_O_3_ using MWE-FC-MLIBS with ceramic micro-laser and helical coil antenna. The standard emission without the microwaves using the same laser energy of 1.0 mJ is shown for comparison.

Prior to the integration of microwaves into the micro-laser, the antenna parameters were optimized by changing the antenna angle, microwave power, and reflected microwave power^19–22^. Figure 4 shows the emission spectrum of the laser-induced plasma on pure Gd_2_O_3_ using a delay time of 0 µs, exposure time of 10 ms, and λ/5000 resolution. In the absence of microwaves, the ablation of the 1.0 mJ drastically declined with longer gate delay until almost no emissions were detected as shown in Supplementary Fig. 2. With the addition of microwave energy, the emission intensity increased, and the emission peaks became more apparent. The difference in emission intensity between the two conditions was large. However, the continuum emissions increased drastically as the microwaves enhanced the atomic emissions of Gd I at 409.8 nm and 419.1 nm.

**Figure 5:**
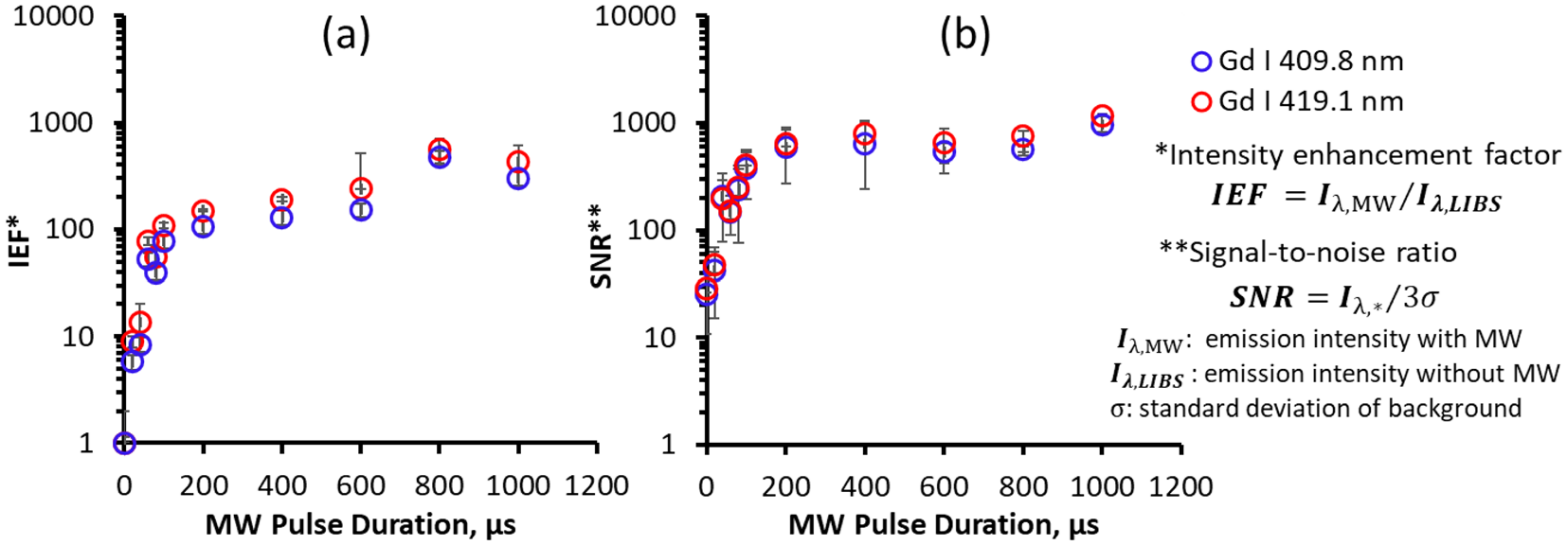
Signal and background variation of Gd I results with microwave (MW) pulse duration. The (**a**) intensity enhancement factor (IEF) and (**b**) signal-to-noise ratio (SNR) of the atomic emission, Gd I, using MWE-FC-MLIBS for increasing microwave pulse duration. The microwave power of 1.0 kW was kept constant.

The optimal impact of microwaves on intensity and SNR was measured using 1.5 µs gate delay, as shown in Fig. 5a and b. The IEF and SNR were calculated based on the Gd I emissions at 409.8 nm and 419.1 nm, with a constant MW power of 1.0 kW and varying MW pulse durations. In the absence of microwaves, the IEF and SNR values were recorded as 1 and 28, respectively, and plotted on the zero value of the x-axis.

It is important to note that the impact of microwaves on the IEF and SNR may vary for other gate delays. For example, the IEF fluctuates between 6 and 20 over the range of 0–200 ns gate delay, while still maintaining a high SNR of over 500. At longer gate delays, the microwave-enhanced emission signals have a greater effect than the rise in background noise, leading to high recorded IEF and SNR values.

Longer microwave pulse durations results in improved emission intensities while maintaining low background noise, as shown by the increase in IEF and SNR. This was due to the temporal increase in microwave absorption into the laser-induced plasma. In high-level radiation environment, laser transmission can be severely attenuated, making it important to collect as much emission as possible for precise quantitative analysis. Therefore, using microwaves to enhance the emission intensities and reduce background noise provides a significant advantage in in-core detection in a nuclear power station.

### Detection of Gd in surrogate nuclear debris

**Figure 6:**
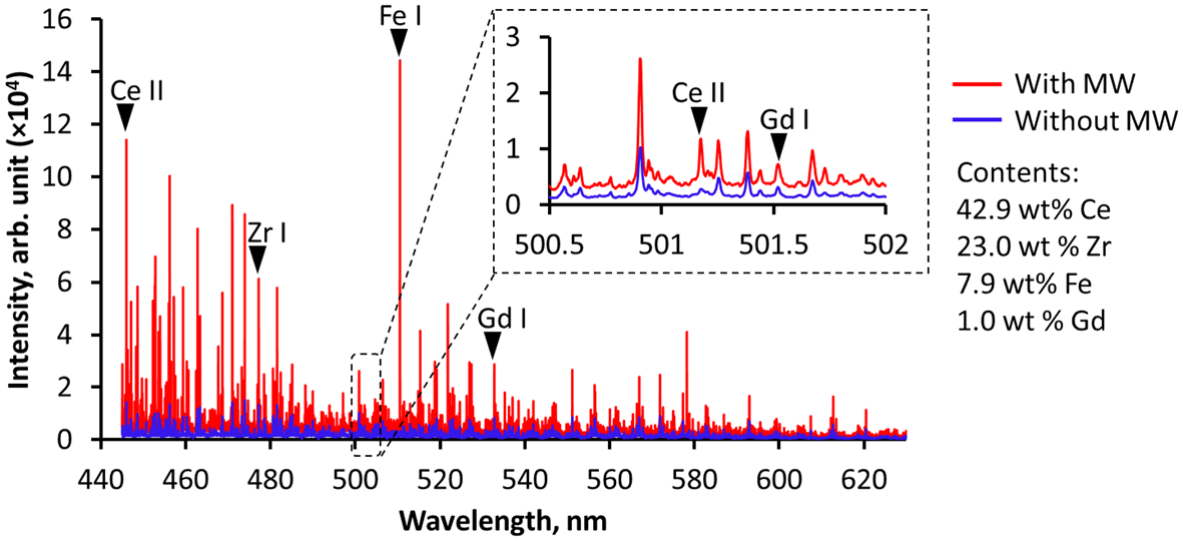
Emission spectra of surrogate nuclear debris (powder mixtures of Gd_2_O_3_, CeO_2_, Zr_2_O_3_, and Fe_2_O_3_) with 1.0 wt% Gd_2_O_3_ using MWE-FC-MLIBS. The standard emission without the microwaves using the same laser energy of 1.0 mJ is shown for comparison.

Detection of Gd in surrogate nuclear debris with 1.0 wt% Gd_2_O_3_ was performed using 1.0 mJ of the micro-laser for varied conditions, with and without 1.0 kW microwaves. Figure 6 shows the emission spectra using an exposure time of 1.0 ms, delay time of 0.5 µs, and resolution of λ/50,000. The microwaves multiplied the emission intensities of the atomic emissions of Ce II (446.0 nm), Zr I (477.2 nm), Fe I (510.6), and Gd I (532.8 nm). At wavelength region 500.5–502 nm, the detection of the ionic emission of Ce II at 501.1 nm, and the atomic emission of Gd I at 501.5 nm were more pronounced with the added microwave energy. However, the microwaves also increased the zero base of the spectrum. Without the added microwave energy, the zero base of the spectrum also fluctuates which generally affects the accuracy of the measurements.

**Figure 7:**
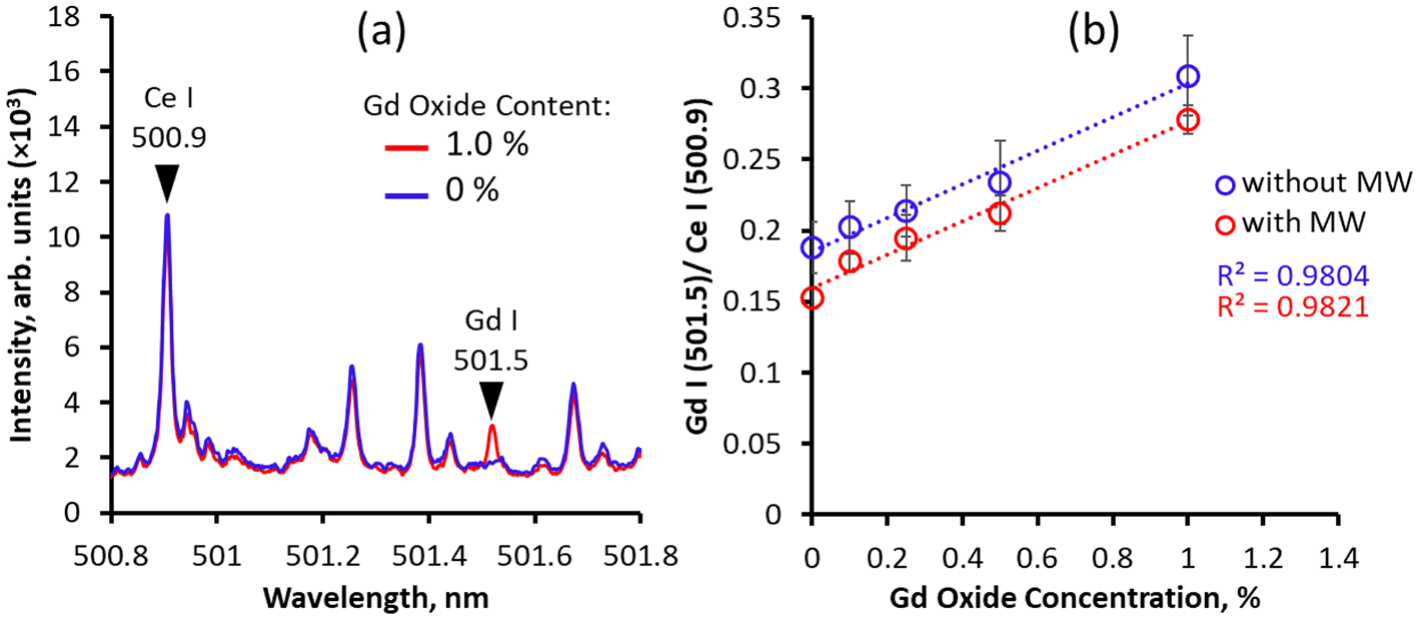
Detection limit of Gd. The (**a**) emission spectra and (**b**) calibration curves of limit of detection of Gd in surrogate nuclear debris.

Figure 7a shows the emission spectra for different concentrations of Gd_2_O_3_ in surrogate nuclear debris. This wavelength region was chosen as self-absorption of Gd emission was less observed. The atomic Gd emission is absent at 0 wt% concentrations but becomes apparent at 1.0 wt%. At 1.0 mJ laser energy, the calibration curves for the determination of the LOD of Gd are shown in Fig. 7b. The ratios of Gd I and Ce I increased with increasing concentration of Gd_2_O_3_. The intensity ratios decreased using the 1.0 kW, 1.0 ms microwaves. The LOD values are shown in Table 1, where the slopes of the calibration curves are approximately equal. The similar slopes indicate that the microwaves did not cause self-absorption of Gd. The microwaves decreased the standard deviation of the intensity ratios of Gd and Ce. The LOD of Gd at 0.459 wt% was larger than that of previously reported measurements at 0.060 wt% using 10 mJ and 8 ns^33^. However, the microwaves decreased the LOD value by 60%, resulting in a detection limit of 0.187 wt%.

**Table 1:** Limit of detection (LOD) of Gd in surrogate nuclear debris using MWE-FC-MLIBS with ceramic micro-laser and helical coil antenna.

	Without MW	With MW
Slope (S)	0.118	0.117
St. Dev. Gd ($\sigma$)	0.018	0.007
LOD (wt%)*	0.459 ± 0.070	0.187 ± 0.012

Without the microwaves, the LOD is also shown.

*Limit of detection (LOD) = 3*σ*/*S*.

*σ*: standard deviation of Gd/Ce at 0% Gd.

*S*: calibration slope.

## Discussions

Significant progress in the in-core detection of nuclear debris inside the Fukushima Daiichi Nuclear Power Station using LIBS has led to the development of FC-LIBS^5,7,9^ and FC-MLIBS^9,34^. The radiation-induced attenuation of light transmission from 580 to 640 nm limits the application of FC-LIBS. As a low-cost alternative, FC-MLIBS uses a disposable low-cost ceramic micro-laser and simultaneously resolves the radiation-induced attenuation problem because the 808 nm wavelength used for end-pumping is not affected. In addition, radiation-deteriorated ceramics have been effectively recovered by thermal treatment. However, the collected emissions may still be attenuated by the required long optical fibers. Moreover, the laser power of FC-MLIBS is limited to a maximum of 10 mJ as the ceramics deteriorate with longer exposure to gamma-ray radiation. Microwaves can potentially solve the attenuation issues in in-core detection applications of nuclear debris as they can be applied remotely to enhance the laser-induced plasma.

The development of a microwave source and helical coil antenna for MWE-LIBS applications on alumina has been reported previously^22^. The effects of microwaves on the laser-induced ablation of Gd_2_O_3_ using standard LIBS in a reduced-pressure environment have been discussed in reference^15^. The detection of Gd in surrogate nuclear debris using FC-MLIBS has also been reported previously^4^. However, there are no previous reports on the effects of microwaves on micro-laser ablation. In this study, we evaluated the effects of microwaves on the analysis of Gd using MWE-FC-MLIBS under atmospheric pressure conditions in open ambient air. This is also the first analytical application of the developed helical antenna.

The effects of microwaves on the plasma dynamics of the laser-induced ablation of Gd_2_O_3_ using a micro-laser have been qualitatively discussed. Delayed absorption of microwaves due to the shielding effects of high-density ablation has been observed. The propagation of microwaves into the plasma increases with the relaxation of the plasma resulting to expansion of the plasma and sustained emission which lasted more than the microwave duration. The plasma dynamics were consistently observed for Gd_2_O_3_ and Al_2_O_3_.

In the spectroscopic analysis, we found that microwaves enhanced the emission intensities of both micro-laser ablated pure Gd_2_O_3_ and surrogate nuclear debris. However, pronounced continuum emissions were observed in low-resolution spectrometers (λ/5000) and the fluctuation of the zero base in high-resolution spectrometers (λ/50,000) persisted. Despite this, the continuum emissions and the zero-base fluctuation with microwave input did not hinder the quantitative detection of Gd in surrogate nuclear debris, as evident by the decrease in the standard deviation of the Gd I/Ce I emission intensity ratio. The use of microwaves also decreased the LOD by 60% when compared with the FC-MLIBS results using 1.0 mJ.

## Conclusions

This study reports the analysis of Gd_2_O_3_, both pure and mixed in surrogate nuclear debris, using MWE-FC-MLIBS with a ceramic micro-laser. In pure Gd_2_O_3_, the expansion of the emitted laser ablation was observed to last until 1200 µs when transmitting 1.0 kW, 1.0 ms microwaves into the ablation area. The laser-induced plasma initially shields the microwaves from interacting with the inner layer. As the impact of the laser energy diminishes, the absorption of microwaves into the plasma increases, accelerating the expansion of the plasma. Beyond 200 µs, the plasma and air interaction intensify as the plasma drifts away from the sample. The plasma exponentially expires after 1.0 ms (after the microwave duration ends). In the spectroscopic analysis of pure Gd_2_O_3_, the IEF and SNR increased logarithmically, which was linked to the temporal increase in microwave absorption into the plasma. Gd detection was possible by increasing the concentrations of Gd_2_O_3_. Calibration curves were constructed from the intensity ratios of Gd and Ce, which had equal slope values for the MWE-FC-MLIBS and standard LIBS conditions. This indicates that self-absorption is not present, even though the zero base of the spectra seems to increase with application of microwaves. The standard deviation of the calibration curves was significantly reduced when microwaves were applied. The detection limit of Gd in surrogate nuclear debris was also decreased by 60% by use of the microwaves, resulting in a detection limit of 0.187 wt%.

## Supplementary Information

Supplementary Figures.

## Data Availability

Data underlying the results presented in this paper are not publicly available at this time but may be obtained from the authors upon reasonable request by contacting Yuji Ikeda at yuji@i-lab.net.
