# Supplementary material for: Analysis of gadolinium oxide using microwave-enhanced fiber-coupled micro-laser-induced breakdown spectroscopy
Source: Sci Rep. 2023 Mar 24;13:4828. doi: 10.1038/s41598-023-32146-x (PMC10039063; doi:10.1038/s41598-023-32146-x)
Supplement: Supplementary file 1 — Supplementary Figures. [file 41598_2023_32146_MOESM1_ESM.docx]

Supplementary Figures


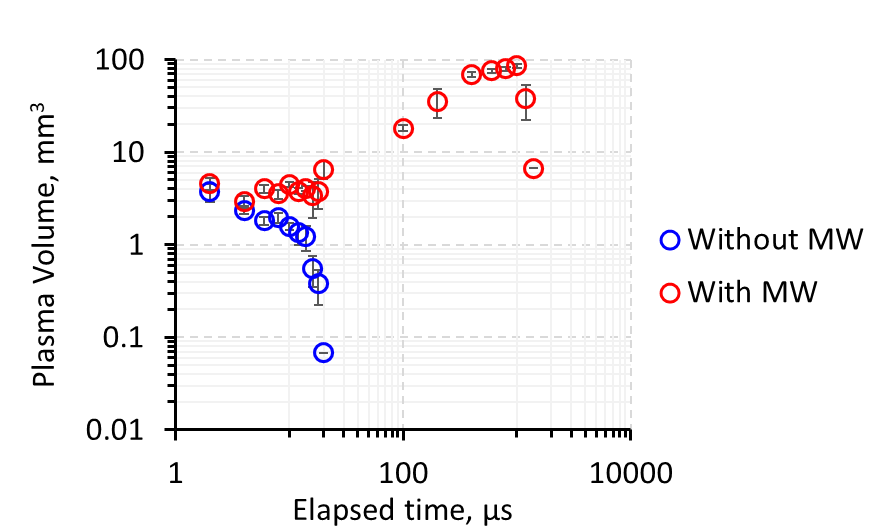


**Supplementary Figure 1**. Temporal variations of the plasma volume in fiber-coupled micro-laser-induced breakdown spectroscopy of pure Gd for varied conditions, without and with MW.


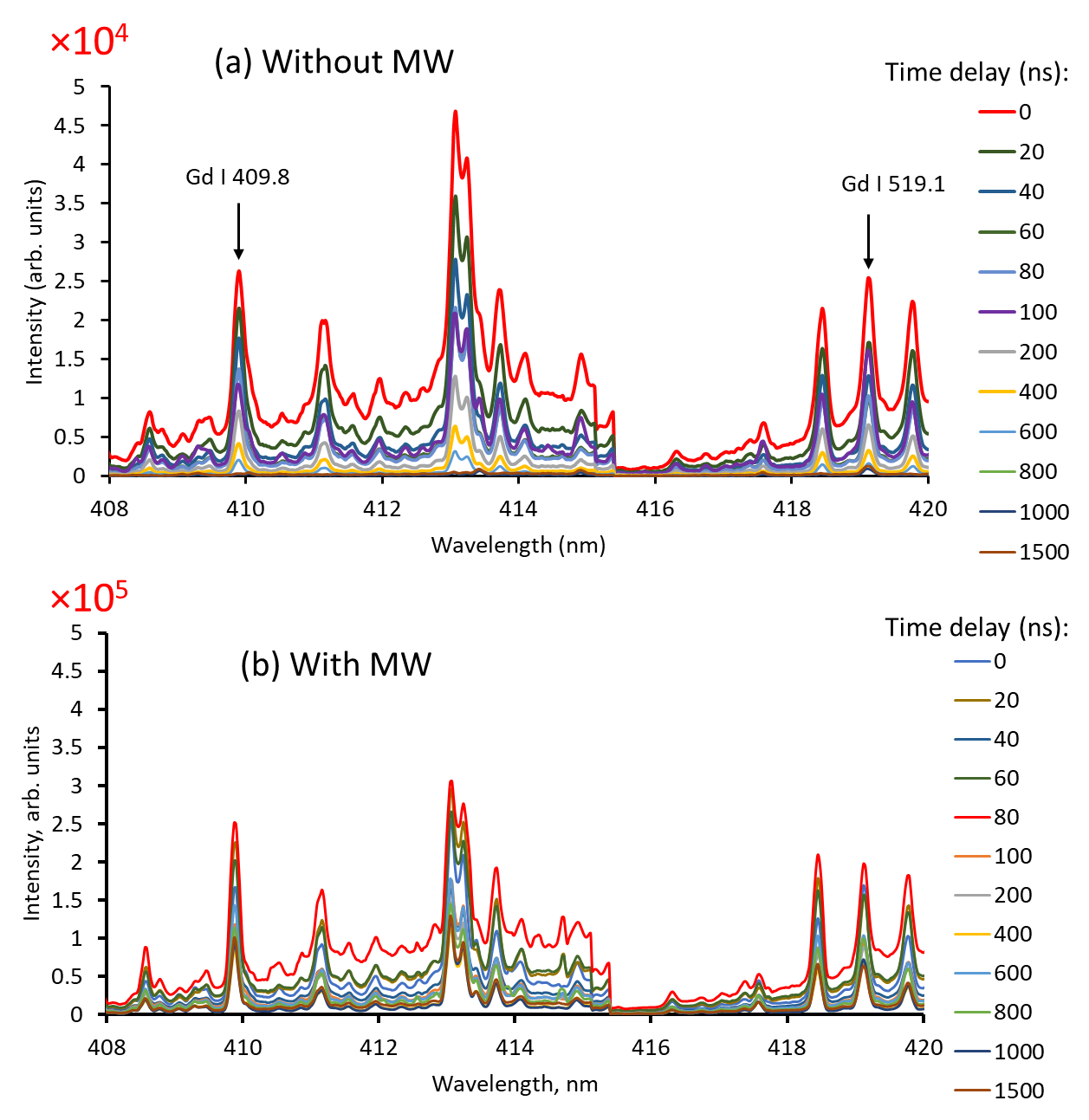


**Supplementary Figure 2**. Effects of microwaves on the emission intensities of Gd in fiber-coupled micro-laser-induced breakdown spectroscopy for varied conditions, (a) without and (b) with MW.
